# Perceived health, musculoskeletal disorders, work conditions and safety climate in relation to patient handling and movement − a multicentre cross-sectional study at healthcare workplaces

**DOI:** 10.1186/s12891-025-09330-3

**Published:** 2025-11-14

**Authors:** Charlotte Wåhlin, Jan Sandqvist, Paul Enthoven, Sebastian Buck, Nadine Karlsson, Emma Nilsing Strid

**Affiliations:** 1 Occupational and Environmental Medicine Centre, Department of Health, Medicine and Caring Sciences, Division of Prevention, Rehabilitation and Community Medicine, Unit of Clinical Medicine, Linköping University, Linköping, Sweden; 2 Clinical Department of Occupational and Environmental Medicine in Linköping, Region Östergötland, Linköping, Sweden; 3 Unit of Intervention and Implementation Research for Worker Health, Institute for Environmental Medicine, Karolinska Institutet, Stockholm, Sweden; 4 Department of Health, Medicine and Caring Sciences, Division of Prevention, Rehabilitation and Community Medicine, Unit of Occupational Therapy, Linköping University, Norrköping, Sweden; 5 Department of Health, Medicine and Caring Sciences, Division of Prevention, Rehabilitation and Community Medicine, Unit of Physiotherapy, Linköping University, Linköping, Sweden; 6 Department of Orthopaedics in Linköping, Region Östergötland, Linköping, Sweden; 7 Department of Health, Medicine and Caring Sciences, Division of Society and Health, Linköping University, Linköping, Sweden; 8 Department of Occupational and Environmental Medicine, Faculty of Medicine and Health, Örebro University, Örebro, Sweden

**Keywords:** Occupational health, Ergonomics, Nursing, Health personnel workers, Safety management, Working environment, Safety culture, Work-related musculoskeletal disorders

## Abstract

**Background:**

A safe healthcare work environment is essential to promote patient safety and healthcare workers’ (HCWs) well-being. Nevertheless, musculoskeletal disorders (MSDs) remain highly prevalent, particularly in relation to patient handling and movement (PHM). This study aims to describe and compare HCWs health, MSDs, working conditions, and safety climate in relation to PHM in hospital care units and nursing homes, as well as to identify desired workplace improvements.

**Methods:**

This multicentre cross-sectional study was based on data collected in 2023 as part of a prospective cluster-randomized trial conducted in Sweden. In total, 1,214 HCWs in 17 hospital care units and 27 nursing homes completed a questionnaire assessing health, PHM routines, organizational conditions, and safety climate using the NOSACQ-50 instrument. Factor analyses of PHM and work environment variables informed mixed linear regression models, evaluating associations with perceived safety climate. Qualitative content analysis was performed on open-ended responses regarding desired workplace improvements.

**Results:**

MSDs were highly prevalent, with 79% of HCWs reporting symptoms in the past week. Nursing home HCWs experienced more pain sites, particularly in extremities. Compared to nursing home HCWs, hospital care HCWs more frequently disagreed that PHM activities were guided by clear policies, written guidelines, established routines, or regular training (*p* < 0.001). Hospital HCWs demonstrated lower adherence to safety policies and less systematic risk assessment. Despite these challenges, the overall perceived safety climate was moderately positive, with NOSACQ-50 mean scores ranging from 3.1 to 3.6. Over 40% of HCWs reported significant physical work environment issues, including heavy lifting, repetitive movements, uncomfortable positions, and high work pace. Regression analyses revealed that organizational support, clear guidelines, ergonomic and psychosocial work factors, and especially managerial support (*p* < 0.001), were strongly associated with higher perceived safety climate. Qualitative data mostly underscored needs for improved staffing, education, structured routines, and better access to assistive equipment, particularly ceiling lifts.

**Conclusions:**

Substantial physical and organizational challenges related to PHM were reported by HCWs across both care contexts. The findings suggest that interventions emphasizing leadership engagement, a systematic approach to training, and tailored solutions are essential to strengthen the safety climate and reduce HCWs’ health risks.

**Trial registration:**

NCT05276180 Registration date 02112022.

**Supplementary Information:**

The online version contains supplementary material available at 10.1186/s12891-025-09330-3.

## Background

 Creating a safe care and work environment for patients and healthcare workers (HCWs) relies on effective systematic management of both patient safety and organizational safety. When organizations hold collective beliefs, values, and behaviours prioritizing and supporting safety, a strong safety culture can be built, contributing to safety for both patients and HCWs [1, 2]. However, there is still a lack of safety for patients and HCWs, with numerous risk factors for potential incidents and injuries [3, 4]. There is a call for support of the healthcare workforce to provide safe care for patients, as there is no patient safety without HCW safety [5, 6]. The primary causes of work-related musculoskeletal disorders (WMSDs) among HCWs stem from high physical and mental workload, staffing shortages, and limited influence over work conditions, as well as lack of support from colleagues and lack of availability of assistive devices for safe patient handling and movement (PHM) [7, 8]. WMSDs among HCWs are linked to several factors, including years of experience in direct patient care, management strategies promoting early mobilization, low job satisfaction, and a lower perception that management cares about safety [9]. WMSDs may occur during repetitive work tasks or in awkward postures such as when performing PHM [3, 8, 10]. Notably, WMSDs arising from PHM rank among the most prevalent injuries. These injuries predominantly affect the lumbar spine, neck, and shoulders, and are most frequent among nurses and assistant nurses [9, 11, 12].

Recent reviews of epidemiological studies report a high annual prevalence rate of WMSDs among nurses, with rates up to 77.2% [12]. However, none of the studies included in the reviews were conducted in a Scandinavian country where HCWs are employed within regional healthcare services and municipalities, such as in hospital care units and nursing homes. In Sweden, healthcare is provided by 21 regions and 290 municipalities, with different geographical and organizational prerequisites [13]. In order to prevent WMSDs among HCWs and promote safety in relation to PHM routines, risks assessments and appropriate multifactorial workplace interventions are suggested [8, 14]. Equipment availability, ceiling lift availability, supervisor encouragement, and annual training increase HCWs’ use of equipment for PHM [9, 14, 15]. Perceptions of organizational safety practice are important as high perceptions are associated with lower physical workload and WMSDs [16]. Moreover, ensuring the safety of PHM requires that both managers and HCWs prioritize and adhere to safety practices. Several standards and guidelines on safe PHM support the importance of establishing a culture of safety and implementing practices for risk assessment, training and maintaining competence [17, 18]. In a recent study, the Swedish version of the risk assessment instrument TilThermometer was found feasible for assessing risks of physical exposure among HCWs in relation to PHM [19]. However, it is unclear if practices for risk assessment and interventions to promote safe PHM are used and prioritized within Swedish hospital care units and nursing homes. Despite many challenges with WMSDs among HCWs, research focusing on HCWs’ perceptions of safe PHM in healthcare workplaces remains scarce.

The aim of the present study was to describe and compare HCWs’ health, MSDs, working conditions and occupational safety climate in relation to PHM in hospital care units and nursing homes, and to examine factors associated with levels of safety climate. A further aim was to identify areas of work environment improvements in relation to PHM.

## Methods

### Study design

This multicentre cross-sectional study is part of a cluster-randomized controlled trial evaluating intervention strategies for safe PHM in the healthcare sector. Detailed information about the research project is provided in the published study protocol [20]. The baseline data of the project were used for the present study. Ethics approval was received from the Swedish National Ethical Board (Dnr 2021–00578). This study adheres to the CONSORT guidelines and includes a completed CONSORT checklist as an additional file (Supplementary Material 1).

### Healthcare setting and study sample

The inclusion criteria for this study were HCWs in the healthcare sector, including hospital care units (regional healthcare sector) and nursing homes (municipal healthcare sector). The hospital care units were inpatient care units with a minimum of 15 HCWs employed at the unit. The nursing homes were units for older adults with a minimum of 15 HCWs employed at the unit. The care units were located in different parts of Sweden in larger, mid-sized and small cities. The exclusion criteria for the study were care units providing outpatient home nursing, paediatric care, parts of emergency care and psychiatric care.

In Sweden, healthcare services are primarily administered by regional and local authorities (municipalities). Care is delivered within hospital settings as well as in municipal care environments, each characterized by distinct organizational structures, differences in the demographics of care recipients, and varying levels of available resources and support. A major distinction between the two settings is that hospital care patients typically have shorter stays focused on acute care, whereas elderly individuals in municipal care often reside long-term in nursing homes. Including both types of care units − hospital care units and nursing homes − and comparing them with regard to safety climate and the health status of HCWs may provide valuable insights.

### Recruitment and data collection

Information about the study and recruitment of workplaces in hospital care units and nursing homes took place between 2022-10-01 and 2023-01-15. This was done through contact people such as managers and HR representatives, as well as via various communication channels, including union representatives, social media, and newsletters. Care units who expressed interest received detailed information about the study and its procedures from the research team. Managers shared the information with HCWs through emails and workplace meetings. Before officially joining the study, the manager at each care unit signed an informed written consent.

All participants received oral information about the study from their managers and written information from the research group. HCWs gave their informed consent prior to answering the questionnaire. The questionnaire was digitally distributed to HCWs at participating units between February and March 2023. Reminders were sent out by email once a week. A description of the questions and items included in the questionnaire is provided below.

### Questionnaire

Contextual factors were collected from each participating care unit at baseline concerning type of care unit and staffing levels. The questionnaire answered by the HCWs contained questions about individual factors, health and musculoskeletal and work-related disorders, work ability, work performance and sick leave, participation in patient handling and movement (PHM), work conditions, safety climate, and open questions concerning HCWs’ suggestions on work environment improvements.

### Individual characteristics

Participants answered questions on their age, sex, profession, and years at their current workplace.

### Health, musculoskeletal and work-related disorders

General self-reported health was obtained by the question: “In general, would you say your health is…?”, with a five-point ordinal rating scale ranging between excellent and poor [21]. Other questions concerned musculoskeletal pain and symptoms from the neck, shoulders, elbows, wrists/hands, back, and lower extremities. The participants were asked about the prevalence of pain in these locations during the previous 12 months, and in the previous seven days [22]. They reported if they had problems related to mental or physical exposure at work: “Do you experience problems related to the load and work tasks that you perform in your current job?” with response options “yes” or “no”. If yes, a list was presented where one or more alternatives could be specified: neck, shoulders, elbows, wrists/hands, thoracic spine, lumbar spine, hips, knees, ankles/feet, headache, stress-related problems, depression, and other with an open answer option.

### Work ability, work performance, presenteeism and sick leave

HCWs responded to the single item “current work ability compared with life-time best” on an 11-point ordinal rating scale from 0 to 10 [23]. The work ability of the HCWs was also assessed in relation to physical and mental demands at work based on the work ability index using a 5-point Likert scale, ranging from 1 “Very poor” to 5 “Very good” (WAI) [24]. Furthermore, HCWs were asked whether they had experienced any work environment problems affecting health and work performance and sickness presence (presenteeism) in the previous seven days [25, 26]. Participants were asked whether their illness or injury constituted a hindrance in their current work. Seven response options were available to capture the extent of perceived work limitations, ranging from no hindrance to complete inability to work. In previous studies, self-reported sickness absence has been shown to have acceptable reliability [27]. One question focused on sickness absence: “How many days have you been absent from work due to illness or injury in the past year?” with the rating alternatives: no days, 1–7 days; 8–12 days; 25–99 days; 100–365 days.

### Patient handling and movement (PHM)

The frequency of PHM was evaluated based on questions from a previous study [7]: “How many patients do you PHM per day on a regular working day?” with the rating alternatives: [1] none; [2] one day a week; [3] 2–3 days per week; [4] 4 days per day; [5] every working day; [6] 1–5 times per day; [7] 6–10 times per day; [8] 11–20 times per day; [12] 21 or more times per day. The same scale was also used to evaluate (1) how often a transfer is performed together with a colleague, and (2) how often transfer was performed without using the aids needed to perform it in a safe way. Patient mobility independence was assessed by asking about the.

proportion of patients able to do transfers independently and without using aids/equipment during the past week, with five statements rating from “No patients” to “All patients”. The Borg-CR 10 scale was used [28, 29] specifically for reporting work strain in relation to PHM and participants reported how often PHM was performed each day. Furthermore, 14 research questions were used to assess the safety climate in relation to PHM, previously published in a Swedish guideline [17]. These questions involved the dimensions of using equipment, other aspects of safety, performing risk assessment, and collaboration between caregivers and patients. Responses were rated on a 4-point ordinal scale ranging from 1 (Strongly disagree) to 4 (Strongly agree) (Supplementary Material 2).

### Work conditions

Part of the Structured Multidisciplinary Work Environment Survey (SMET) [30] was used. It contains questions about physically, environmentally and psychosocially demanding work tasks. The answers from the SMET questionnaire are trichotomized [1–10] and categorized as 1–3 = low degree of problems, 4–7 = some degree of problems, and 8–10 = high degree of problems. The questionnaire also included a question on work conditions regarding change of workload over time, where responses were rated on a 3-point ordinal scale (1 = decreased; 2 = unchanged; 3 = increased).

### Safety climate

The safety climate was measured using NOSACQ-50, a questionnaire that has been validated in various contexts [31]. This questionnaire comprises 50 items across seven safety climate dimensions in the workplace: (1) management safety priority, commitment and competence; (2) management safety empowerment; (3) management safety justice; (4) workers’ safety commitment; (5) workers’ safety priority and risk non-acceptance; (6) safety communication, learning and trust in co-workers’ safety competence; and (7) trust in the efficacy of safety system. The questionnaire is scored on a 4-point ordinal rating scale, ranging from 1 (“strongly disagree”) to 4 (“strongly agree”). The score for each dimension consists of the mean value of the included items, where a score greater than 3.30 on each dimension indicates a good level for safety climate and the need for continuing safety development. A total score for NOSACQ was calculated as the mean of NOSACQ dimensions 1 to 7. The total score has been used and presented in previous research [32].

### HCWs’ suggestion for work environment improvements

HCWs responded to three open-ended questions designed to gather suggestions for workplace improvements: (1) “Do you have suggestions on how your work environment and aspects of safety can be improved at your workplace?”; (2) “Do you have suggestions on work methods, equipment, or assistive devices that could facilitate tasks related to patient transfers?”; and (3) “Do you have other suggestions regarding patient handling and movement?”.

### Statistical analysis

Descriptive data were presented with mean and SD, median and range, or counts and percentages. The independent t-test was used for comparisons of continuous variables between groups. Mann-Whitney U-test were used for comparison between groups of variables on an ordinal scale. Differences in proportions between groups were examined with chi-squared tests or Fisher’s exact test. Comparative analysis was made in relation to safety climate, working conditions, and type of organization/care unit.

The NOSACQ-50 total score was used as the primary outcome (dependent variable) of the regression analyses. A mixed linear regression model was used to account for the fact that individuals are nested within healthcare units. In a first step, a factor analysis was performed to derive simplified dimensions of measured aspects of Safety PHM and SMET, respectively. The extraction method was principal component analysis, and an orthogonal rotation was performed with varimax and Kaiser normalization [33]. Factors not considered relevant to the research question were not included in the regression analysis. For each identified relevant dimension, the factor scores were computed for each respondent and were used as independent variables in subsequent regression analysis. Taking into account that individuals are nested within healthcare units, a mixed linear regression was used to examine associations between NOSACQ total and the derived dimensions of Safety PHM and SMET (occupational characteristics), age (individual characteristic), and manager support (SMET28 item 3). A level of 5% was considered to be statistically significant. The quantitative data were analysed using IBM SPSS version 29 and Stata 18.

Responses to the three open-ended questions on suggestions for work environmental improvements were systematically analysed with a summative and manifest approach to qualitative content analysis [34].This implies analyzing for the appearance of a particular word or content in textual material, a quantification to explore usage instead of inferring meaning. Initially, all responses were carefully read to identify recurring areas for improvement. First, categories were developed inductively, each representing a distinct aspect of workplace improvement in relation to PHM, such as working routines, safety practices, and equipment suggestions. Thereafter text contents were systematically coded into the categories, followed by a quantification of the distribution of responses across the categories. CW developed the categories, and CW together with PE coded the data, which were subsequently discussed and agreed upon with ENS and JS.

## Results

### Individual characteristics

In total, 1,214 HCWs answered the baseline questionnaire, of which 510 worked at 17 hospital care units, and 704 worked at 27 nursing homes; see Table 1. Participants working at hospital care units had a lower mean (SD) age (40.7 (12.7)) than those working at nursing homes (47.9 (11.9)). Most participants were women, with a higher proportion of men working at hospital care units (13.7%, *n* = 70) than at nursing homes (8.4%, *n* = 59), *p* = 0.005. Most participants were either assistant nurses (69.6%) or nurses (17.8%), with a lower proportion of assistant nurses and a higher proportion of nurses working at hospital care units compared to nursing homes (50.2% versus 83.7%, and 39.0% versus 2.4%, respectively). Participants from hospital care units more often worked 76% to full-time compared to participants from nursing homes (93.4% versus 85.1%, respectively, *p* < 0.001). Participants from hospital care units had more years at their current workplace than those working at nursing homes (*p* < 0.001).

**Table 1:** Individual characteristics, health, musculoskeletal and work-related disorders of health care workers (HCWs). Comparison between hospital care and nursing home HCWs

Items/question	Total all HCWs *n* = 1 214		Hospital Care units HCWs *n* = 510		Nursing homes HCWs, *n* = 704		*P*-value*
**Care units**	44		17		27		
**Age in years**, mean (SD)	44.9	(12.8)	40.7	(12.7)	47.9	(11.9)	**< 0.001**^**t**^
Range	20–68		20–70		20–70		
**Sex**,** n (%)**							**0.005**^**F**^
Male	129	(10.6)	70	(13.7)	59	(8.4)	
Female	1081	(89.0)	439	(86.1)	642	(91.2)	
Prefer not to say	4	(0.3)	1	(0.2)	3	(0.4)	
**Profession**,** n (%)**							**< 0.001**
Assistant nurse	845	(69.6)	256	(50.2)	589	(83.7)	
Nurse	216	(17.8)	199	(39.0)	17	(2.4)	
Care assistant	62	(5.1)	1	(0.2)	61	(8.7)	
Occupational-/Physical therapist	58	(4.8)	34	(6.7)	24	(3.4)	
Other	33	(2.7)	20	(3.9)	13	(1.8)	
**Years at workplace**,** n (%)**							**< 0.001**
< 2 years	316	(26.0)	178	(34.9)	138	(19.6)	
< 5 years	259	(21.3)	125	(24.5)	134	(19.0)	
5–10 years	268	(22.1)	84	(16.5)	184	(26.1)	
>10 years	371	(30.6)	123	(24.1)	248	(35.2)	
**General health**							**< 0.001**^**M**^
Excellent	90	(7.4)	51	(10.0)	39	(5.5)	
Very good	363	(29.9)	181	(35.5)	182	(25.9)	
Good	533	(43.9)	197	(38.6)	336	(47.7)	
Fair	209	(17.2)	76	(14.9)	133	(18.9)	
Bad	19	(1.6)	5	(1.0)	14	(2.0)	
**During the past 12 months**,** have you had…….**							
Musculoskeletal pain, yes	1138	(93.7)	475	(93.1)	663	(94.2)	0.461
number of locations, mean (SD)	4.4	(2.4)	4.1	(2.3)	4.6	(2.5)	**< 0.001**^**M**^
Neck pain	853	(70.3)	364	(71.4)	489	(69.5)	0.472
Shoulder, elbow and/or wrist/hand pain	947	(78.0)	378	(74.1)	569	(80.8)	**0.005**
Lumbar spine pain	901	(74.2)	372	(72.9)	529	(75.1)	0.387
Hip, knee and/or ankle/foot pain	787	(64.8)	311	(61.0)	476	(67.6)	**0.017**
**During the past 7 days**,** have you had…….**							
Musculoskeletal pain, yes	975	(78.8)	396	(77.6)	561	(79.7)	0.390
number of locations, mean (SD)	2.7	(2.3)	2.4	(2.1)	3.0	(2.5)	**< 0.001**^**M**^
Neck pain	496	(40.9)	203	(39.8)	293	(41.6)	0.525
Shoulder, elbow and/or wrist/hand pain	656	(54.0)	248	(48.6)	408	(58.0)	**0.001**
Lumbar spine pain	575	(47.4)	228	(44.7)	347	(49.3)	0.114
Hip, knee and/or ankle/foot pain	570	(47.0)	207	(40.6)	363	(51.6)	**< 0.001**
**Mental and/or physical work related problems in current work**							
work-related problems, yes	925	(76.2)	365	(71.6)	560	(79.5)	**0.001**
number of work-related problems	2.1	(1.9)	1.7	(1.6)	2.4	(2.1)	**< 0.001**^**M**^
Neck pain	419	(34.5)	170	(33.3)	249	(35.4)	0.461
Shoulder, elbow and/or wrist/hand pain	624	(51.4)	211	(41.4)	413	(58.7)	**< 0.001**
Low back pain	493	(40.6)	192	(37.6)	301	(42.8)	0.074
Hip, knee and/or ankle/foot pain	410	(33.8)	125	(24.5)	285	(40.5)	**< 0.001**
Headache	313	(25.8)	140	(27.5)	173	(24.6)	0.258
Stress-related problems	347	(28.6)	138	(27.1)	209	(29.7)	0.317
Depression	105	(8.6)	53	(10.4)	52	(7.4)	0.066
Other health problems	61	(5.0)	24	(4.7)	37	(5.3)	0.665

* The p-values show the difference between hospital care and nursing home health care workers (HCWs). The p-values represent Chi-square test p-values, unless stated otherwise. ^t^ independent sample t-test p-value; ^F^ Fisher’s exact test; ^M^ Mann-Whitney U test p-value; bold figures represent p-values < 0.05

### General health, musculoskeletal and work-related disorders

About 80% of all participants reported excellent to good general health, see Table 1. Almost 94% of all participants reported having had musculoskeletal pain during the past 12 months, with participants from hospital care units reporting pain at a lower mean (SD) number of locations than participants from nursing homes (4.1 (2.3) versus 4.6 (2.5), respectively). Pain located in the neck, the lumbar spine, and in the upper or lower extremities was common. There were no significant differences regarding neck or lumbar spine pain during the past 12 months between the groups, while participants from hospital care units less often had pain in the upper and lower extremities than participants from nursing homes (74.1% and 61% versus 80.8% and 67.6%, respectively).

Almost 80% of all participants reported musculoskeletal pain during the past seven days, with participants from hospital care units reporting pain at a lower mean (SD) number of locations than participants from nursing homes (2.4 (2.1) versus 3.0 (2.5), respectively, *p* < 0.001). Again, pain located in the neck, the lumbar spine, and in the upper or lower extremities was common. There were no significant differences regarding neck or lumbar spine pain during the past seven days between the groups, while participants from hospital care units less often had pain in the upper and lower extremities than participants from nursing homes (48.6% and 40.6% versus 58% and 51.6%, respectively, *p* < 0.001).

The percentage and number of problems related to physical or mental workload was lower in participants working in hospital care units than those working in nursing homes (71.6% and 1.6 versus 79.5% and 2.1, respectively, *p* ≤ 0.001). Besides musculoskeletal problems, headache (25.8%), stress related problems (28.6%) and depression (8.6%) were problems related to participants’ mental and/or physical workload.

### Work ability, work performance and sick leave

About three-quarters of the participants (76.3%) reported their current work ability to be very good or good in relation to the physical demands, and 73.1% reported their current work ability to be excellent or good in relation to the mental demands of their work, see Table 2. Participants working at hospital care units more often reported that they did not have an illness or injury or that these were an obstacle in their current work compared to participants from nursing homes (66.1% versus 41.3%, *p* = 0.003–0.006). Fewer participants working at hospital care units than those working at nursing homes reported they could do their work but get symptoms (35.9% versus 45.3%, respectively). In total 28.3% of participants reported that they had been on sick leave for eight days or more during the past 12 months. Almost half of the participants (41.4%) had experienced health problems during the past seven days but still chose to go to work. There were no significant differences in sick leave or experiencing health problems but still going to work between participants at hospital care units or nursing homes.

**Table 2:** Work ability, work performance and sick leave. Comparison between hospital care and nursing home health care workers (HCWs)

Items/question	Total all HCWs *n* = 1 214		Hospital Care units HCWs *n* = 510		Nursing homes HCWs *n* = 704		*P*-value*
	n	(%)	n	(%)	n	(%)	
**Work ability**							
Work ability now compared to highest ever work ability, 0–10	8.6	(1.8)	8.7	(1.7)	8.4	(1.8)	0.018^M^
How do you assess your current work ability in relation to the physical demands of your work?							**< 0.001**^**M**^
Very good	296	(24.4)	161	(31.6)	135	(19.2)	
Good	630	(51.9)	255	(50.0)	375	(53.3)	
Moderate	245	(20.2)	80	(15.7)	165	(23.4)	
Bad or very bad	43	(3.5)	14	(2.7)	29	(4.1)	
How do you assess your current ability to work in relation to the mental demands your work makes?							**0.013**
Very good	279	(23.0)	134	(26.3)	145	(20.6)	
Good	608	(50.1)	261	(51.2)	347	(49.3)	
Moderate	269	(22.2)	96	(18.8)	173	(24.6)	
Bad or very bad	58	(4.8)	19	(3.7)	39	(5.5)	
**Fit for work 1–7**							
**Is your illness or injury an obstacle in your current work?**							
There are no obstacles	437	(36.0)	208	(40.8)	229	(32.5)	**0.003**
I have no illnesses or injuries	261	(21.5)	129	(25.3)	132	(18.8)	**0.006**
I can do my job, but get symptoms	502	(41.4)	183	(35.9)	319	(45.3)	**< 0.001**
I sometimes have to reduce the pace of work or change the way I work	148	(12.2)	50	(9.8)	98	(13.9)	**0.030**
I often have to reduce the pace of work or change the way I work	35	(2.9)	9	(1.8)	26	(3.7)	**0.047**
Due to my illness or injury, I can only work part-time	51	(4.2)	19	(3.7)	32	(4.5)	0.482
In my own opinion, I am completely unable to work	4	(0.3)	0	(0.0)	4	(0.6)	0.088
**Work environment and sickness absence/presence**							
How many days have you been away from work due to illness or injury (care, treatment or examination) in the past year?							**0.071**^**M**^
None	438	(36.1)	195	(38.2)	243	(34.5)	
1–7 days	433	(35.7)	183	(35.9)	250	(35.5)	
8–24 days	246	(20.3)	99	(19.4)	147	(20.9)	
25–99 days	79	(6.5)	29	(5.7)	50	(7.1)	
100–365 days	18	(1.5)	4	(0.8)	14	(2.0)	
During the past seven days, have you experienced problems (physical, mental, social) in your work environment? (yes)	479	(39.5)	200	(39.2)	279	(39.6)	0.884
Have you experienced health problems in the past seven days but still chose to go to work? (yes)	503	(41.4)	195	(38.2)	308	(43.8)	0.054
	Mean	SD	Mean	SD	Mean	SD	
During the past seven days, to what extent did work environment-related problems affect your work performance ^a^	3.6	(2.6)	3.7	(2.5)	3.6	(2.6)	0.431^M^
During the past seven days, to what extent did health problems affect your work performance ^b^	3.1	(2.5)	3.0	(2.4)	3.3	(2.6)	0.069^M^

^a^ Effect on work performance by work environment related problems score 0 “work environment problems have not affected my work performance” to 10 “work environment problems completely prevented me from working”

^b^ Effect on work performance by health problems score 0 “health problems have not affected my work performance” to 10 “health problems completely prevented me from working”

* The p-values show the difference between hospital care and nursing home health care workers (HCWs). The p-values represent Chi-square test p-values, unless stated otherwise. ^M^ Mann-Whitney U test p-value; bold figures represent p-values < 0.05

### Patient handling and movement (PHM)

Many participants (82.2%) participated at least every working day in PHM, while 25.9% did so between six to more than 20 times daily; see Table 3. Participants working at hospital care units less often reported that they carried out patient transfers with a colleague every working day than participants from nursing homes (67.6% versus 80.8%, respectively, *p* < 0.001). In total, 78% of participants carried out PHM without using aids/equipment up to four days per week, while 22% did so at least every working day. Some participants (3.6%) carried out PHM without using aids/equipment six to more than 20 times per day.

**Table 3:** Work task and performance in patient handling and movement (PHM). Comparison between hospital care and nursing home health care workers (HCWs)

Items/question	Total all HCWs *n* = 1 214		Hospital Care units HCWs *n* = 510		Nursing homes HCWs *n* = 704		*P*-value*
	*n*	(%)	*n*	(%)	*n*	(%)	
PHM							
How often do you participate in patient transfers?							**0.044**^**M**^
Never to 1 day per week	86	(7.1)	46	(9.0)	40	(5.7)	
2–4 days per week	130	(10.7)	72	(14.1)	58	(8.3)	
Every working day	539	(44.5)	194	(38.0)	345	(49.1)	
1–5 times daily	143	(11.8)	75	(14.7)	68	(9.7)	
6–10 times daily	152	(12.5)	67	(13.1)	85	(12.1)	
11–20 times daily	116	(9.6)	41	(8.0)	75	(10.7)	
>20 times daily	46	(3.8)	15	(2.9)	31	(4.4)	
How often do you carry out patient transfers with a colleague?							**< 0.001**^**M**^
Never to 1 day per week	126	(10.4)	57	(11.2)	69	(9.8)	
2–4 days per week	174	(14.4)	108	(21.2)	66	(9.4)	
Every working day	543	(44.8)	196	(38.5)	347	(49.4)	
1–5 times daily	141	(11.6)	60	(11.8)	81	(11.5)	
6–10 times daily	138	(11.4)	61	(12.0)	77	(11.0)	
11–20 times daily	67	(5.5)	24	(4.7)	43	(6.1)	
>20 times daily	22	(1.8)	3	(0.6)	19	(2.7)	
How often do you carry out patient transfers without using aids/equipment?							0.559^M^
Never to 1 day per week	866	(71.5)	355	(69.7)	511	(72.8)	
2–4 days per week	79	(6.5)	54	(10.6)	25	(3.6)	
Every working day	182	(15.0)	63	(12.4)	119	(17.0)	
1–5 times daily	41	(3.4)	21	(4.1)	20	(2.8)	
6–10 times daily	25	(2.1)	10	(2.0)	15	(2.1)	
11–20 times daily	13	(1.1)	5	(1.0)	8	(1.1)	
>20 times daily	5	0.4)	1	(0.2)	4	(0.6)	
Proportion of patients able to do transfers independently and without using aids/equipment during past week							**< 0.001**^**M**^
No patients/care recipient	133	(11.0)	40	(7.9)	93	(13.2)	
Some patients/care recipients, about one quarter.	623	(51.4)	242	(47.5)	381	(54.3)	
Half of the patients/care recipients	267	(22.0)	140	(27.5)	127	(18.1)	
Many patients/care recipients (about three quarters)	156	(12.9)	79	(15.5)	77	(11.0)	
All patients/care recipients.	32	(2.6)	8	(1.6)	24	(3.4)	
**Work strain**							
	Mean	SD	Mean	SD	Mean	SD	
How do you estimate the physical effort you generally experience during patient transfers ^a^	6.1	(2.3)	5.9	(2.1)	6.2	(2.4)	0.063^M^

^a^ Score 0 “none” to 10 “extremely large”

* The p-values show the difference between hospital care and nursing homes health care workers (HCW). ^M^ Mann-Whitney U test p-value; bold figures represent p-values < 0.05

### Safety in relation to PHM

Most participants agreed or strongly agreed that they made an effort together to carry out safe PHM (94.6%) and had access to work equipment and aids (97.1%), but only 74.9% reported that there is a fixed ceiling lift where needed; see Table 4. Participants working at hospital care units strongly disagreed or disagreed that their work was based on a work environment policy, a written guideline, established routines or some specific method more often than participants from nursing homes (*p* < 0.001). Participants working at hospital care units more often strongly disagreed or disagreed that before carrying out PHM they assessed the patient’s health and function, the ability to move themselves, and the need for more healthcare workers to do the transfer, than participants from nursing homes (*p* < 0.001). Furthermore, participants working at hospital care units more often strongly disagreed or disagreed that they regularly conducted training in PHM and discussed how to prevent work-related injuries during PHM, than participants from nursing homes (*p* < 0.001).

**Table 4:** Safety in relation to patient handling and movement (PHM). Comparison between hospital care and nursing home health care workers (HCWs)

	Total all HCWs *n* = 1 214		Hospital Care units HCWs *n* = 510		Nursing homes HCWs *n* = 704		*P*-value*
	n	%	n	%	n	%	
**PHM**							
**1. We who work here make an effort together to carry out safe PHM**							**0.002**^**M**^
Strongly disagree	14	(1.2)	3	(0.6)	11	(1.6)	
Disagree	52	(4.3)	29	(5.7)	23	(3.3)	
Agree	516	(42.5)	239	(46.9)	277	(39.3)	
Strongly agree	632	(52.1)	239	(46.9)	393	(55.8)	
**2. At our workplace**,** we have access to work equipment and aids that we can use when working with PHM**							**0.003**^**M**^
Strongly disagree	6	(0.5)	1	(0.2)	5	(0.7)	
Disagree	29	(2.4)	13	(2.5)	16	(2.3)	
Agree	351	(28.9)	173	(33.9)	178	(25.3)	
Strongly agree	827	(68.2)	323	(63.3)	504	(71.7)	
**3. At our workplace there is a fixed ceiling lift where needed**							0.067^M^
Strongly disagree	223	(18.4)	39	(7.6)	184	(26.2)	
Disagree	82	(6.8)	55	(10.8)	27	(3.8)	
Agree	263	(21.7)	154	(30.2)	109	(15.5)	
Strongly agree	645	(53.2)	262	(51.4)	383	(54.5)	
**4. We who work here take joint responsibility for the use of work equipment and aids when working with PHM**							**0.001**^**M**^
Strongly disagree	10	(0.8)	5	(1.0)	5	(0.7)	
Disagree	53	(4.4)	29	(5.7)	24	(3.4)	
Agree	389	(32.1)	184	(36.1)	205	(29.2)	
Strongly agree	761	(62.7)	292	(57.3)	469	(66.7)	
**5. At our workplace**,** we work based on a work environment policy**							**< 0.001**^**M**^
Strongly disagree	35	(2.9)	17	(3.3)	18	(2.6)	
Disagree	89	(7.3)	47	(9.2)	42	(6.0)	
Agree	464	(38.3)	223	(43.7)	241	(34.3)	
Strongly agree	624	(51.5)	223	(43.7)	401	(57.1)	
**6. At our workplace**,** we work based on a written guideline for the PHM**							**< 0.001**^**M**^
Strongly disagree	73	(6.0)	53	(10.4)	20	(2.8)	
Disagree	172	(14.2)	121	(23.7)	51	(7.3)	
Agree	415	(34.2)	204	(40.0)	211	(30.1)	
Strongly agree	552	(45.5)	132	(25.9)	420	(59.8)	
**7. At our workplace**,** we work based on an established routine for how to carry out risk assessments when working with PHM**							**< 0.001**^**M**^
Strongly disagree	99	(8.2)	66	(12.9)	33	(4.7)	
Disagree	186	(15.3)	110	(21.6)	76	(10.8)	
Agree	415	(34.2)	198	(38.8)	217	(30.9)	
Strongly agree	512	(42.2)	136	(26.7)	376	(53.6)	
**8. Before we do a PHM we carry out a risk assessment according to some specific method**							
Strongly disagree	206	(17.0)	131	(25.7)	75	(10.7)	
Disagree	236	(19.5)	141	(27.6)	95	(13.5)	
Agree	376	(31.0)	139	(27.3)	237	(33.8)	
Strongly agree	394	(32.5)	99	(19.4)	295	(42.0)	
**9. Before we carry out PHM**,** we assess the health and functional capacity of the patient/care recipient**							**< 0.001**^**M**^
Strongly disagree	18	(1.5)	7	(1.4)	11	(1.6)	
Disagree	55	(4.5)	27	(5.3)	28	(4.0)	
Agree	332	(27.4)	174	(34.1)	158	(22.5)	
Strongly agree	807	(66.6)	302	(59.2)	505	(71.9)	
**10. Before we do a PHM**,** we assess the patient/care recipient’s risk of falling**							**< 0.001**^**M**^
Strongly disagree	15	(1.2)	3	(0.6)	12	(1.7)	
Disagree	45	(3.7)	26	(5.1)	19	(2.7)	
Agree	300	(24.8)	166	(32.5)	134	(19.1)	
Strongly agree	852	(70.3)	315	(61.8)	537	(76.5)	
**11. Before we carry out PHM**,** we assess the patient’s/care recipient’s ability to move themselve**							**< 0.001**^**M**^
Strongly disagree	13	(1.1)	3	(0.6)	10	(1.4)	
Disagree	49	(4.0)	18	(3.5)	31	(4.4)	
Agree	322	(26.6)	175	(34.3)	147	(20.9)	
Strongly agree	828	(68.3)	314	(61.6)	514	(73.2)	
**12. Before we carry out PHM**,** we assess whether we need more healthcare workers to be able to transfer the patient**							0.110^M^
Strongly disagree	15	(1.2)	2	(0.4)	13	(1.9)	
Disagree	30	(2.5)	10	(2.0)	20	(2.8)	
Agree	288	(23.8)	143	(28.0)	145	(20.7)	
Strongly agree	879	(72.5)	355	(69.6)	524	(74.6)	
**13. At our workplace**,** we regularly conduct training in the area of PHM and transfer techniques**							**< 0.001**^**M**^
Strongly disagree	190	(15.7)	109	(21.4)	81	(11.5)	
Disagree	285	(23.5)	146	(28.6)	139	(19.8)	
Agree	345	(28.5)	138	(27.1)	207	(29.5)	
Strongly agree	392	(32.3)	117	(22.9)	275	(39.2)	
**14. At our workplace**,** we regularly discuss how we can prevent work-related injuries during PHM**							**< 0.001**^**M**^
Strongly disagree	140	(11.6)	82	(16.1)	58	(8.3)	
Disagree	293	(24.2)	165	(32.4)	128	(18.2)	
Agree	417	(34.4)	173	(33.9)	244	(34.8)	
Strongly agree	362	(29.9)	90	(17.6)	272	(38.7)	

* The p-values show the difference between hospital care and nursing homes health care workers(HCW), bold figures represent p-values < 0.05. ^M^ Mann-Whitney U test p-value; bold figures represent p-values < 0.05

### Work conditions

About 60% of participants reported that the workload had increased since they started working at their current workplace; see Table 5. A considerable number of participants experienced a high degree of problems in their work associated with the physical work environment; these included: heavy lifting *n* = 535 (44.1%), repetitive movements *n* = 401 (33%), unilateral or fixed working positions *n* = 299 (24.6%), uncomfortable working positions *n* = 454 (37.4%), a high work-pace *n* = 438 (36.1%), and narrow spaces *n* = 317 (26.1%); see Table 5. The item responsibilities, rights and/or expectations had the lowest number (%) of participants experiencing a high degree of problems, *n* = 116 (9.6%), and the item anxiety about not having time to complete your work had the highest number *n* = 241 (19.9%).

**Table 5:** Workload and work conditions (SMET). Comparison between hospital care and nursing home health care workers (HCWs)

Items/question	Total all HCWs *n* = 1 214		Hospital Care units HCWs *n* = 510		Nursing homes HCWs *n* = 704		*P*-value*
	n	%	n	%	n	%	
**How have your work conditions regarding workload changed at this workplace since you started?**							0.317
Decreased	74	(6.1)	28	(5.5)	46	(6.5)	
Unchanged	438	(36.1)	196	(38.4)	242	(34.4)	
Increased	701	(57.8)	286	(56.1)	415	(59.0)	
**SMET**							
**Physical work environment** Do you, in your work, experience any problems associated with.							
Physical work environment							
Heavy lifting?							0.373
low degree of problems	221	(18.2)	95	(18.6)	126	(17.9)	
some degree of problems	458	(37.7)	202	(39.6)	256	(36.4)	
high degree of problems	535	(44.1)	213	(41.8)	322	(45.7)	
Repetitive movements?							**0.002**
low degree of problems	325	(26.8)	154	(30.2)	171	(24.3)	
some degree of problems	488	(40.2)	215	(42.2)	273	(38.8)	
high degree of problems	401	(33.0)	141	(27.6)	260	(36.9)	
Unilateral or fixed working positions?							0.192
low degree of problems	404	(33.3)	180	(35.3)	224	(31.8)	
some degree of problems	511	(42.1)	217	(42.5)	294	(41.8)	
high degree of problems	299	(24.6)	113	(22.2)	186	(26.4)	
Uncomfortable working positions?							0.501
low degree of problems	282	(23.2)	127	(24.9)	155	(22.0)	
some degree of problems	478	(39.4)	196	(38.4)	282	(40.1)	
high degree of problems	454	(37.4)	187	(36.7)	267	(37.9)	
A high work-pace?							**0.028**
low degree of problems	292	(24.1)	104	(20.4)	188	(26.7)	
some degree of problems	484	(39.9)	207	(40.6)	277	39.3)	
high degree of problems	438	(36.1)	199	(39.0)	239	33.9)	
Narrow spaces? Do you, in your work, experience any problems associated with.							**< 0.001**
low degree of problems	547	(45.1)	194	(38.0)	353	(50.1)	
some degree of problems	350	(28.8)	127	(24.9)	223	(31.7)	
high degree of problems	317	(26.1)	189	(37.1)	128	(18.2)	
**Organisational and social factors Do you**,** in your work**,** experience any problems associated with.**							
Work routines and the distribution of tasks?							0.085
low degree of problems	575	(47.4)	251	(49.2)	324	(46.0)	
some degree of problems	449	(37.0)	193	(37.8)	256	(36.4)	
high degree of problems	190	(15.7)	66	(12.9)	124	(17.6)	
Collaboration, communication and feedback?							**0.009**
low degree of problems	620	(51.1)	279	(54.7)	341	(48.4)	
some degree of problems	419	(34.5)	175	(34.3)	244	(34.7)	
high degree of problems	175	(14.4)	56	(11.0)	119	(16.9)	
Support from your boss/employer?							**< 0.001**
low degree of problems	790	(65.1)	363	(71.2)	427	(60.7)	
some degree of problems	274	(22.6)	101	(19.8)	173	(24.6)	
high degree of problems	150	(12.4)	46	(9.0)	104	(14.8)	
Responsibilities, rights and/or expectations?							**0.008**
low degree of problems	738	(60.8)	334	(65.5)	404	(57.4)	
some degree of problems	360	(29.7)	139	(27.3)	221	(31.4)	
high degree of problems	116	(9.6)	37	(7.3)	79	(11.2)	
Your possibilities to develop in your work?							**< 0.001**
low degree of problems	766	(63.1)	359	(70.4)	407	(57.8)	
some degree of problems	324	(26.7)	115	(22.5)	209	(29.7)	
high degree of problems	124	(10.2)	36	(7.1)	88	(12.5)	
Unreasonable demands?							**< 0.001**
low degree of problems	692	(57.0)	327	(64.1)	365	(51.8)	
some degree of problems	352	(29.0)	134	(26.3)	218	(31.0)	
high degree of problems	170	(14.0)	49	(9.6)	121	(17.2)	
Having control and being able to handle the psychological demands that arise?							**< 0.001**
low degree of problems	631	(52.0)	297	(58.2)	334	(47.4)	
some degree of problems	389	(32.0)	159	(31.2)	230	(32.7)	
high degree of problems	194	(16.0)	54	(10.6)	140	(19.9)	
Having no time to take breaks on an ordinary working day?							**0.012**
low degree of problems	661	(54.4)	256	(50.2)	405	(57.5)	
some degree of problems	369	(30.4)	178	(34.9)	191	(27.1)	
high degree of problems	184	(15.2)	76	(14.9)	108	(15.3)	
Anxiety about making serious mistakes?							**0.007**
low degree of problems	773	(63.7)	326	(63.9)	447	(63.6)	
some degree of problems	288	(23.7)	136	(26.7)	152	(21.6)	
high degree of problems	152	(12.5)	48	(9.4)	104	(14.8)	
Anxiety about not having time to complete your work?							**0.002**
low degree of problems	579	(47.7)	237	(46.5)	342	(48.6)	
some degree of problems	393	(32.4)	190	(37.3)	203	(28.9)	
high degree of problems	241	(19.9)	83	(16.3)	158	(22.5)	

Abbreviations: SMET, Structured Multidisciplinary work Evaluation Tool

* The p-values show the difference between hospital care and nursing home health care workers (HCWs). The p-values represent Chi-square test p-values; bold figures represent p-values < 0.05

Regarding the physical work environment, participants working at hospital care units less often experienced a high degree of problems for the items repetitive movements (*p* = 0.002), uncomfortable working positions (*p* = 0.028), and more often a high degree of problems associated with narrow spaces. Regarding organizational and social factors, there were significant differences between participants working at hospital care units and nursing homes for all items, except for the item work routines and the distribution of tasks. Participants working at hospital care units less often experienced a high degree of problems for the items collaboration, communication and feedback (*p* = 0.009), support from your manager/employer (*p* < 0.001), responsibilities, rights and/or expectations (*p* = 0.008), your opportunities to develop in your work (*p* < 0.001), unreasonable demands (*p* < 0.001), having control and being able to handle the psychological demands that arise (*p* < 0.001), having no time to take breaks on an ordinary working day (*p* < 0.001), anxiety about making serious mistakes (*p* = 0.007), and anxiety about not having time to complete your work (*p* = 0.002).

### Safety climate

The safety climate mean (SD) NOSACQ-50 scores across seven dimensions ranged from 3.1 (0.6) to 3.6 (0.4); see Table 6. Dimension 3 − management safety justice, dimension 6 − safety communication, learning and trust in co-workers’ safety competence, and dimension 7 − trust in the efficacy of safety system, reached a mean value of 3.5 or higher. There were small but significant differences in dimensions 3, 4, 5, 6 and 7 between participants from hospital care units and nursing homes.

**Table 6:** Safety climate table. Comparison between hospital care and nursing home health care workers (HCWs)

Items/question	Total all HCWs *n* = 1 214		Hospital Care units HCWs *n* = 510		Nursing homes HCWs *n* = 704		*P*-value*
	Mean	(SD)	Mean	(SD)	Mean	(SD)	
**NOSACQ-50** ^**a**^							
Dimension 1 management safety priority, commitment and competence:	3.3	(0.5)	3.3	(0.5)	3.3	(0.5)	0.215^M^
Dimension 2 management safety empowerment:	3.3	(0.5)	3.3	(0.5)	3.3	(0.6)	0.313^M^
Dimension 3 Management safety justice:	3.5	(0.5)	3.5	(0.5)	3.4	(0.6)	**0.041**^**M**^
Dimension 4 Workers’ safety commitment:	3.4	(0.5)	3.4	(0.5)	3.5	(0.6)	**0.026**^**M**^
Dimension 5 Workers safety priority and risk non-acceptance:	3.1	(0.6)	3.2	(0.5)	3.1	(0.6)	**0.030**^**M**^
Dimension 6 safety communication, learning and trust in co-workers’ safety competence	3.5	(0.5)	3.4	(0.5)	3.5	(0.5)	**0.002**^**M**^
Dimension 7 trust in the efficacy of safety system	3.6	(0.4)	3.6	(0.4)	3.6	(0.5)	**0.008**^**M**^
NOSACQ-50 total mean	3.4	(0.4)	3.4	(0.4)	3.4	(0.4)	0.837^M^

^a^ NOSACQ, Nordic safety climate questionnaire

* The p-values show the difference between hospital care and nursing home health care workers (HCWs), ^M^ Mann-Whitney U test p-values; bold figures represent p-values < 0.05

### Factors associated with perceived level of safety climate

The factor analysis for Safety PHM resulted in three factors or dimensions that together explained 61% of the variance. The dimensions were Safety PHM1 education and guidelines (explaining 41% of variance), Safety PHM2 health and function of patients (explaining 12% of variance), and Safety PHM3 equipment and cooperation (explaining 8% of variance); The factor analysis for selected items of SMET resulted in five factors/dimensions that together explained 60% of the variance. The dimensions were SMET1 organization and cooperation (explaining 34% of variance), SMET2 ergonomics and physical strain (explaining 9% of variance), SMET3 psychological safety (explaining 7% of variance), and SMET4 physical work environment (explaining 5% of variance). The fifth dimension, sitting and eyesight demands (explaining 5% of variance), was considered not relevant to the research question and not included in the regression analysis. All three dimensions derived from the Safety PHM were positively associated with a higher perceived NOSACQ-50 safety climate for PHM (*p* < 0.001) (Table 7). For all four dimensions derived from the SMET, less problems were associated with a higher perceived NOSACQ-50 safety climate for PHM (*p* < 0.001–0.005). Furthermore, positive manager support was associated with a higher perceived NOSACQ-50 safety climate for PHM (*p* < 0.001). The model was adjusted for age in categories.

**Table 7:** Mixed linear regression of the associations between NOSACQ-50 total (dependent variable) and Safety PHM and SMET dimensions (independent variables)

	**Model**^a^	
	**β (CI 95%)**	**P-value**
**Safety PHM dimensions**		
Education and guideline	0.06 (0.04-0.07)	<.001
Health and function of patients	0.10 (0.08-0.11)	<.001
Equipment and cooperation	0.10 (0.08-0.12)	<.001
**SMET dimensions**		
Organization and cooperation	0.11 (0.10 - 0.13)	<.001
Ergonomics and physical strain	0.02 (0.01 -0.04)	0.005
Psychological safety	0.08 (0.06 - 0.10)	<.001
Physical work environment	0.04 (0.02 -0.06)	<.001
Positive Manager support*	0.21 (0.13- 0.29)	<.001

Abbreviations: β, regression coefficient; CI 95%, Confidence Interval

^a^Model adjusted for all variables in the table, Safety PHM dimensions, SMET dimensions, Positive manager support and age

### HCWs’ suggestions for work environment improvements

A total of 878 (72.4%) HCWs provided a response to at least one of the three open-ended questions, of which 615 (70%) gave two or more suggestions. The responses were systematically coded into nine categories, reflecting different areas of suggested work environment improvements regarding PHM. The categories were: (1) knowledge, education and training (*n* = 267, providing 306 suggestions); (2) communication and collaboration (*n* = 137, providing 150 suggestions); (3) facilities (*n* = 134, providing 150 suggestions); (4) routines, safety procedures, and policies (*n* = 277, providing 326 suggestions); (5) staffing and scheduling (*n* = 268, providing 307 suggestions); (6) equipment, aids for patient transfer, and ergonomics (*n* = 280, providing 324 suggestions); (7) ceiling lifts (*n* = 144, providing 168 suggestions); (8) satisfied with current conditions (*n* = 62, no suggestions for change); and (9) other work environment improvements (*n* = 263, providing 292 suggestions). Other work environment improvements included, amongst others, cooperation with other HCPs (occupation therapist, physiotherapist, physician), support from managers, and a general demand for better work conditions. The most frequently mentioned suggestions related to needs for improved staffing, education and training, structured routines, and better access to assistive equipment, particularly ceiling lifts.

## Discussion

The findings in this study reveal concurrent high prevalence of MSDs and high self-rated work ability, indicating that presenteeism is common among HCWs in both hospital care units and nursing homes. The need for targeted work environment improvements was further highlighted by the inconsistent and diverse application of safe PHM practices and organizational shortcomings. Differences in PHM procedures, particularly in hospital settings, underscore critical areas for intervention. HCWs proposed a range of measures to promote safer PHM. Key priorities among HCWs in both hospital care units and nursing homes included ensuring adequate staffing levels, rotating physically demanding tasks to reduce strain, improving knowledge with education and practical training and the planning and coordination of patient transfers. Finally, the findings underscore the critical impact of leadership for safe work practices, as positive managerial support was the strongest predictor of high safety climate in relation to PHM.

The findings of this study reveal a complex interplay between work ability, musculoskeletal pain, and work performance. While most participants rated their work ability as good or excellent, musculoskeletal pain was highly prevalent, affecting nearly 94% in the past year, and with almost 80% of participants reporting recent pain during the past seven days. Pain in the neck, lumbar spine, and extremities was common in both care settings. Notably, nursing home HCWs were significantly older and they reported more frequent extremity pain than hospital HCWs, suggesting differences in physical strain related to setting and task characteristics.

Both a higher number of patient transfers and older age has been reported to be associated with an increased risk of work related MSDs among nurses [36].The risk of low back pain has been shown to increase with the frequency of patient transfers [7], which may indicate that physical strain during PHM could be one important contributing factor to the high prevalence of MSDs among HCWs. Previous research has also highlighted MSDs as highly prevalent among HCWs globally and the causes are multifactorial involving aspect of physical, psychosocial, organizational and individual factors [8, 11, 12, 37]. In a study, comparing workers in construction and healthcare, those with higher aerobic capacity and lower body mass index tended to report lower levels of musculoskeletal pain despite being exposed to high occupational physical workloads [38]. The majority of the participants in our study described their work as both mentally and physically demanding. These findings can be related to previous research among HCWs in nursing homes, which demonstrated that low decision authority, poor leadership, high quantitative demands, a fast work pace, and low organizational justice were associated with an increased risk of reporting higher levels of physical exertion [37]. The HCWs, particularly those in nursing homes, also reported other work-related concerns such as headaches, stress, and depressive symptoms. These results are in line with Swedish national data pointing to decreasing mental health in caring occupations [39], and similar trends have been reported in the US, where a high prevalence of depression among healthcare workers has been observed [40]. According to a report published by the Swedish Work Environment Authority, 55% of women in health and social care report excessive workload and limited opportunities for recovery [39].

Nearly one-third of the participants in our study reported taking sick leave exceeding eight days in the past year. Additionally, almost half of participants reported health problems during the previous week but still chose to work. Although nursing home HCWs more frequently reported working despite health problems compared to hospital HCWs, no significant differences were found between settings regarding overall sick leave or presenteeism. This pattern suggests that working despite pain may be a normalized behaviour across settings, embedded within workplace culture, as demonstrated in previous research [41]. While presenteeism may reflect commitment or perceived necessity, its potential impact on performance, injury risk, and patient safety warrants attention. Presenteeism has been linked to HCWs’ sense of duty and consideration for colleagues and managers, and is often driven by high levels of commitment [42]. It is also associated with impaired performance, higher injury risk, and compromised patient safety, as well as broader impacts on health, well-being, and economic outcomes [43]. A study by Dahini et al. [44] shows that lower levels of presenteeism are linked to favourable organizational factors, specifically, high leadership ratings and adequate staffing resources. Variations in PHM procedures, especially within hospital settings, highlight key areas where improvements are needed. In response, HCWs in this study proposed a range of measures to promote safer PHM. Key priorities included ensuring adequate staffing levels, rotating physically demanding tasks to reduce strain, and improving the planning and coordination of patient transfers. Organizational and situational factors have previously been shown to significantly influence the increased use of assistive devices, with care situations playing a particularly critical role in nursing homes [45]. In this study, even though PHM tasks were performed frequently, often multiple times per shift, many participants reported conducting transfers without assistive devices, and collaborative handling was less common in hospital settings compared to nursing homes. In line with these quantitative findings, the open-ended responses further highlighted that the availability and use of assistive devices were considered key priorities among HCWs in both hospital care units and nursing homes. The availability of equipment and ceiling lifts, along with supervisor encouragement and annual training, have previously all been associated with higher utilization of safe PHM equipment [9]. Previous research shows that the use of motorized assistive devices significantly reduces work-related MSDs [46]. Moreover, the safest methods for patient handling involve the use of floor or ceiling lifts, as well as air-assisted devices for lateral transfers and repositioning tasks [10, 15]. The presence of organizational shortcomings, lack off staff and increasing workload hinders the consistent application of safe practices using assistive devices [47, 48]. In the present study, organizational shortcomings were particularly evident in hospital settings. Staff reported an absence of formal work environment policies, established routines, and regular PHM training. Although systematic patient assessments of mobility and health prior to transfers are endorsed in the scientific literature and in Swedish guidelines and regulatory frameworks [17, 19], they were infrequently performed in our study. The findings in the current study suggest possible weaknesses in local safety climate, especially in relation to leadership, procedural clarity, and opportunities for competence development for improving PHM. Addressing these gaps through multifactorial workplace interventions with clear guidelines, available equipment, and annual training, along with supervisor encouragement is essential to promote safe PHM practices [8, 9, 14]. Lee et al. [16] found that organizations actively fostering a positive safety climate, implementing ergonomic measures, and cultivating a people-oriented culture achieved significantly better outcomes. These included lower physical workload and job strain, increased job satisfaction, reduced risk perception, and fewer reports of work-related injuries and symptoms.

The regression model in our study identified several factors significantly associated with a higher perceived safety climate in relation to PHM. These included education and access to guidelines, systematic assessment of patients’ health and functional abilities, appropriate equipment, staff collaboration, organizational and managerial support, ergonomic practices, psychological safety, and the physical work environment. Among these, positive managerial support emerged as the factor with the highest loading, highlighting the pivotal role of leadership in shaping safety perceptions within healthcare environments. Consistent with the present findings, previous studies have also reported that HCWs rated their safety climate positively, with leadership quality and job satisfaction identified as key contributing factors [49, 50]. Further research is needed in the healthcare settings to deepen the understanding of how safety climate can be strengthened in relation to PHM.

### Strengths and limitations

A strength of this study was the large number of participating HCWs from both hospital care units and municipal nursing homes across different regions in Sweden, which enhances the generalizability of the findings. Given that the challenges faced by HCWs are similar across many Western countries, the insights gained may be transferable to comparable settings internationally. The included healthcare units were participants in a cluster randomized trial [20], which may indicate a selection bias towards healthcare units interested in PHM and safety climate. Validated self-reported outcome measures were used. Using the NOSACQ-50 to compare safety climate between hospital healthcare units and nursing homes revealed only minor differences. These results needs to be further explored with inclusion of additional factors such as age, years of employment at the current workplace and staffing levels. HCWs completed the questionnaire through a secure web-based platform which enabled easy access for the responders. A strength of the study was the use of both qualitative and quantitative data where the open-ended questions confirmed the overall findings and also added new information providing a more in-depth and concrete understanding of the results. Varying degree of HCWs exposure to PHM tasks may have influenced the findings. Future studies could benefit from focusing specifically on those HCWs most directly engaged in these work tasks. The survey was in Swedish, which may have excluded participants with limited language skills in Swedish. To explore factors associated with the perceived level of safety climate, factor analyses were initially performed on the Safety PHM and SMET questionnaires. This approach was used to reduce the number of variables in the regression model and to avoid multicollinearity. All identified dimensions demonstrated significant associations with the perceived safety climate in the regression analysis. However, the contribution of individual items within these dimensions was not assessed, which may limit the precision of the findings. Moreover, as the identified dimensions were derived from the present sample, their applicability to other healthcare settings should be interpreted with caution.

## Conclusions

Healthcare workers (HCWs) in both hospital care units and nursing homes reported a paradoxical status, with a high prevalence of MSDs coexisting with high self-rated work ability. Despite frequent task of patient handling and movement (PHM), adherence to safe PHM practices was inconsistent, particularly in hospital settings where organizational shortcomings such as lack of guidelines, insufficient training, and limited use of assistive devices were more evident. Across both settings, HCWs emphasized the importance of adequate staffing, task rotation, improved training, and access to appropriate equipment to reduce strain and improve safety. The HCWs suggestions on work environmental improvements underscore the importance of multifactorial, organizational-level interventions, emphasizing leadership engagement, structured training approaches and context-specific solutions. Addressing these areas is critical to improving healthcare workers’ occupational safety and health in relation to PHM.

## Supplementary Information

Supplementary material 1.

Supplementary material 2.

## Data Availability

The data are not publicly available but can be obtained from the corresponding author on reasonable request.
