# Supplementary material for: Perceived health, musculoskeletal disorders, work conditions and safety climate in relation to patient handling and movement − a multicentre cross-sectional study at healthcare workplaces
Source: BMC Musculoskelet Disord. 2025 Nov 14;26:1048. doi: 10.1186/s12891-025-09330-3 (PMC12619508; doi:10.1186/s12891-025-09330-3)
Supplement: Supplementary file 1 — Supplementary material 1. [file 12891_2025_9330_MOESM1_ESM.docx]

|  | Section/topic | No | CONSORT 2025 checklist item description | Reported on page no. |
| --- | --- | --- | --- | --- |
|  | **Title and abstract** | | |  |
|  | Title and structured abstract | 1a | Identification as a randomised trial  Title: Perceived health, musculoskeletal disorders, work conditions and safety climate in relation to patient handling and movement − a multicentre cross-sectional study at healthcare workplaces.  1a) A multicentre cross-sectional design embedded within a multi-centre randomised controlled trial. This is reported in the title and abstract. | p. 1-2 |
|  |  | 1b | Structured summary of the trial design, methods, results, and conclusions  1b) The abstract contains a structured summary of the trial design, methods, results, and conclusions | p. 2 |
|  | **Open science** | | |  |
|  | Trial registration | 2 | Name of trial registry, identifying number (with URL) and date of registration  2)Trial registration: NCT05276180: <https://clinicaltrials.gov/study/NCT05276180>  Registration date: 02-11-2022 | p. 2 |
|  | Protocol and statistical analysis plan | 3 | Where the trial protocol and statistical analysis plan can be accessed  3) The trial protocol for the RCT can be assessed in the published study protocol. This cross-sectional study is based on baseline data from the RCT; Wåhlin C, Buck S, Sandqvist J, Enthoven P, Fock J, Andreassen M, Strid EN. Evaluation of the implementation and effectiveness of a multifactorial intervention strategy for safe patient handling and movement in the healthcare sector: a study protocol of a cluster randomised controlled trial. BMJ open. 2023;13(2):e067693. This is reported under “Methods – Study design”, and the reference is included in the reference list. | p. 4  References (20)  p. 30 |
|  | Data sharing | 4 | Where and how the individual de-identified participant data (including data dictionary), statistical code and any other materials can be accessed.  4) The data are not publicly available but can be obtained from the corresponding author on reasonable request. | p. 26 |
|  | Funding and conflicts of interest | 5a | Sources of funding and other support (eg, supply of drugs), and role of funders in the design, conduct, analysis and reporting of the trial  5a) This research was funded by AFA Insurance, Sweden, grant number 20240337. | p. 26 |
|  |  | 5b | Financial and other conflicts of interest of the manuscript authors  5b) The authors declare that they have no competing interests. | p. 26 |
|  | **Introduction** | | |  |
|  | Background and rationale | 6 | Scientific background and rationale  6) See page 3-4 | p. 3-4 |
|  | Objectives | 7 | Specific objectives related to benefits and harms  7) See page 4 | p. 4 |
|  | **Methods** | | |  |
|  | Patient and public involvement | 8 | Details of patient or public involvement in the design, conduct and reporting of the trial  8) No patients were included in the study, only healthcare workers employed in healthcare units. See page 5 | p. 5 |
|  | Trial design | 9 | Description of trial design including type of trial (eg, parallel group, crossover), allocation ratio, and framework (eg, superiority, equivalence, non-inferiority, exploratory)  9) This study is a multicentre cross-sectional design embedded within a multi-centre randomised controlled trial. This is reported in the title and abstract and under Methods – Study design. | p. 1  p. 2  p. 4 |
|  | Changes to trial protocol | 10 | Important changes to the trial after it commenced including any outcomes or analyses that were not prespecified, with reason  10) No, in this specific study we only use baseline measurements. | p. 5 |
|  | Trial setting | 11 | Settings (eg, community, hospital) and locations (eg, countries, sites) where the trial was conducted  11)The setting for this study was in the healthcare sector, including hospital care units (regional healthcare sector) and nursing homes (municipal healthcare sector) located in different parts of Sweden. | p. 5 |
|  | Eligibility criteria | 12a | Eligibility criteria for participants  12a) see page 5-6 | p. 5-6 |
|  |  | 12b | If applicable, eligibility criteria for sites and for individuals delivering the interventions (eg, surgeons, physiotherapists)  12b) Not applicable. |  |
|  | Intervention and comparator | 13 | Intervention and comparator with sufficient details to allow replication. If relevant, where additional materials describing the intervention and comparator (eg, intervention manual) can be accessed  13) Not applicable. |  |
|  | Outcomes | 14 | Prespecified primary and secondary outcomes, including the specific measurement variable (eg, systolic blood pressure), analysis metric (eg, change from baseline, final value, time to event), method of aggregation (eg, median, proportion), and time point for each outcome  14) the outcomes/measurements included are described at page 6-9 | p. 6-9 |
|  | Harms | 15 | How harms were defined and assessed (eg, systematically, non-systematically)  15) Not defined for this cross-sectional study |  |
|  | Sample size | 16a | How sample size was determined, including all assumptions supporting the sample size calculation  16 a) The study size was 1 214 participants, of which 510 worked in hospital care units and 704 in nursing homes, see Results page 11 and Table 1. The study size was based on material gathered for the randomized controlled trial in which this study was embedded. The study size was larger than required to do the statistical analyses that were performed for this cross-sectional study. | p. 11-12 |
|  |  | 16b | Explanation of any interim analyses and stopping guidelines  16b) Not applicable |  |
|  | Randomisation: |  |  |  |
|  | Sequence generation | 17a | Who generated the random allocation sequence and the method used  17a) For the RCT a statistician produced a data-generated randomization list, stratifying clusters by organization type (regional inpatient vs. municipal nursing homes). See reference 20 Wåhlin et al. page 30  For the current cross-sectional study random allocation was not applicable. |  |
|  |  | 17b | Type of randomisation and details of any restriction (eg, stratification, blocking and block size)  17b) Inclusion criteria Regional healthcare: inpatient care units with a minimum of 15 HCWs employed at the unit.  Municipal healthcare: nursing homes for older adults with a minimum of 15 HCWs employed at the unit. | p. 5 |
|  |  |  |  | **Reported on page no.** |
|  | Allocation concealment mechanism | 18 | Mechanism used to implement the random allocation sequence (eg, central computer/telephone; sequentially numbered, opaque, sealed containers), describing any steps to conceal the sequence until interventions were assigned  18) A statistician produced a data-generated randomization list, stratifying clusters by organization type (regional inpatient vs. municipal nursing homes). See reference 20 Wåhlin et al. page 30  For the current cross-sectional study random allocation was not applicable. |  |
|  | Implementation | 19 | Whether the personnel who enrolled and those who assigned participants to the interventions had access to the random allocation sequence  19) No, they did not have access |  |
|  | Blinding | 20a | Who was blinded after assignment to interventions (eg, participants, care providers, outcome assessors, data analysts)  20a) Not applicable for this cross-sectional study. |  |
|  |  | 20b | If blinded, how blinding was achieved and description of the similarity of interventions  20b) Not applicable for this cross-sectional study. |  |
|  | Statistical methods | 21a | Statistical methods used to compare groups for primary and secondary outcomes, including harms  21 a) see pages 10-11 | p. 10-11 |
|  |  | 21b | Definition of who is included in each analysis (eg, all randomised participants), and in which group  21 b) see pages 10-11. Also take part of the results pages 11-20 | p. 10-11 and p. 11-20 |
|  |  | 21c | How missing data were handled in the analysis  21c) There were very few missing data because the participants filled in an online questionnaire in which they were “forced” to give a response before they could answer the next question in the questionnaire. |  |
|  |  | 21d | Methods for any additional analyses (eg, subgroup and sensitivity analyses), distinguishing prespecified from post hoc  21d) Not applicable |  |
|  | **Results** | | |  |
|  | Participant flow, including flow diagram | 22a | For each group, the numbers of participants who were randomly assigned, received intended intervention, and were analysed for the primary outcome  22)a Not applicable for this cross-sectional study |  |
|  |  | 22b | For each group, losses and exclusions after randomisation, together with reasons  22b) Not applicable for this cross-sectional study |  |
|  | Recruitment | 23a | Dates defining the periods of recruitment and follow-up for outcomes of benefits and harms  23 a) Recruitment took place between 2022-10-01 and 2023-01-15. |  |
|  |  | 23b | If relevant, why the trial ended or was stopped.  23b) Not relevant |  |
|  | Intervention and comparator delivery | 24a | Intervention and comparator as they were actually administered (eg, where appropriate, who delivered the intervention/comparator, how participants adhered, whether they were delivered as intended (fidelity))  24a) Not applicable for this cross-sectional study |  |
|  |  | 24b | Concomitant care received during the trial for each group  24b) Not applicable for this cross-sectional study |  |
|  | Baseline data | 25 | A table showing baseline demographic and clinical characteristics for each group  25) See pages 11-12 | p.11-12 |
|  | Numbers analysed,  outcomes and estimation | 26 | For each primary and secondary outcome, by group:  ● the number of participants included in the analysis  ● the number of participants with available data at the outcome time point  ● result for each group, and the estimated effect size and its precision (such as 95% confidence interval)  ● for binary outcomes, presentation of both absolute and relative effect size  26) In total for all measurement in this cross-sectional study: 1 214 healthcare workers, see pages 11-12  For statistical methods see pages 10-11. | p. 11-12  p. 10-11 |
|  | Harms | 27 | All harms or unintended events in each group  27)Not applicable for this cross-sectional study |  |
|  | Ancillary analyses | 28 | Any other analyses performed, including subgroup and sensitivity analyses, distinguishing pre-specified from post hoc  28) Not applicable for this cross-sectional study. For statistical analyses see page 10-11. |  |
|  | **Discussion** | | |  |
|  | Interpretation | 29 | Interpretation consistent with results, balancing benefits and harms, and considering other relevant evidence  29) See pages 20-24 | p. 20-24 |
|  | Limitations | 30 | Trial limitations, addressing sources of potential bias, imprecision, generalisability, and, if relevant, multiplicity of analyses  30) see pages 24-25 | p. 24-25 |

Citation: Hopewell S, Chan AW, Collins GS, Hróbjartsson A, Moher D, Schulz KF, et al. CONSORT 2025 Statement: updated guideline for reporting randomised trials. BMJ. 2025; 388:e081123. <https://dx.doi.org/10.1136/bmj-2024-081123>
© 2025 Hopewell et al. This is an Open Access article distributed under the terms of the Creative Commons Attribution License (<https://creativecommons.org/licenses/by/4.0/>), which permits unrestricted use, distribution, and reproduction in any medium, provided the original work is properly cited.

*We strongly recommend reading this statement in conjunction with the CONSORT 2025 Explanation and Elaboration and/or the CONSORT 2025 Expanded Checklist for important clarifications on all the items. We also recommend reading relevant CONSORT extensions. See [www.consort-spirit.org](http://www.consort-spirit.org).
