# Supplementary material for: Perceived health, musculoskeletal disorders, work conditions and safety climate in relation to patient handling and movement − a multicentre cross-sectional study at healthcare workplaces
Source: BMC Musculoskelet Disord. 2025 Nov 14;26:1048. doi: 10.1186/s12891-025-09330-3 (PMC12619508; doi:10.1186/s12891-025-09330-3)
Supplement: Supplementary file 2 — Supplementary material 2. [file 12891_2025_9330_MOESM2_ESM.docx]

**Supplementary material 2**

**Questions related to Patient Handling and Movement**

In the following section, indicate how you perceive that the staff at this workplace manage safety during patient transfers. Only put one X against each question.

|  | Strongly disagree | Disagree | Agree | Strongly agree |
| --- | --- | --- | --- | --- |
| 1. Those of us who work here endeavour to cooperate to carry out safe patient handling and movement. |  |  |  |  |
| 2. At our workplace, we have access to work equipment and aids that we can use for patient handling and movement. |  |  |  |  |
| 3. At our workplace there are fixed ceiling lifts where they are needed. |  |  |  |  |
| 4. Those of us who work here take joint responsibility for work equipment and aids being used for patient handling and movement. |  |  |  |  |
| 5. At our workplace, we work based on a work environment policy. |  |  |  |  |
| 6. At our workplace, we work based on a written guideline or guidance for patient handling and movement. |  |  |  |  |
| 7. At our workplace, we work based on an established procedure for how to conduct risk assessments during patient handling and movement. |  |  |  |  |
| 8. Before we carry out patient handling and movement, we conduct a risk assessment using a specific method |  |  |  |  |
| 9. Before we carry out patient handling and movement, we assess the health and functional capacity of the care recipient. |  |  |  |  |
| 10. Before we carry out patient handling and movement, we assess the care recipient’s risk of a fall. |  |  |  |  |
| 11. Before we carry out patient handling and movement, we assess the care recipient’s ability to move themself. |  |  |  |  |
| 12. Before we carry out patient handling and movement, we assess whether we need more healthcare professionals to be able to transfer the care recipient in a safe way. |  |  |  |  |
| 13. At our workplace, we have regular training in the field of patient handling and movement and transfer knowledge/transfer technology. |  |  |  |  |
| 14. At our workplace, we regularly discuss how we can prevent injuries during patient handling and movement. |  |  |  |  |
|  |  |  |  |  |

Wåhlin C, Sandqvist J, Enthoven P, Buck S, Karlsson N, Nilsing Strid E. Perceived health, musculoskeletal disorders, work conditions and safety climate in relation to patient handling and movement − a multicentre cross-sectional study at healthcare workplaces. *BMC Musculoskeletal disorder*, 2025.
